# Enhanced external counterpulsation modulates the heartbeat evoked potential

**DOI:** 10.3389/fphys.2023.1144073

**Published:** 2023-04-03

**Authors:** Hongyun Liu, Hui Liang, Xiaohua Yu, Guojing Wang, Yi Han, Muyang Yan, Shijun Li, Weidong Wang

**Affiliations:** ^1^ Research Center for Biomedical Engineering, Medical Innovation Research Division, Chinese PLA General Hospital, Beijing, China; ^2^ Key Laboratory of Biomedical Engineering and Translational Medicine, Ministry of Industry and Information Technology, Beijing, China; ^3^ Department of Hyperbaric Oxygen, The First Medical Center, Chinese PLA General Hospital, Beijing, China; ^4^ Department of Diagnostic Radiology, The First Medical Center, Chinese PLA General Hospital, Beijing, China

**Keywords:** enhanced external counterpulsation, heartbeat evoked potential, brain-heart coupling, electroencephalography, electrocardiography, hemodynamics

## Abstract

**Introduction:** Accumulating evidence suggests that enhanced external counterpulsation (EECP) influences cardiac functions, hemodynamic characteristics and cerebral blood flow. However, little is known about whether or how the EECP affects the brain-heart coupling to produce these physiological and functional changes. We aimed to determine if the brain-heart coupling is altered during or after EECP intervention by assessing the heartbeat evoked potential (HEP) in healthy adults.

**Methods:** Based on a random sham-controlled design, simultaneous electroencephalography and electrocardiography signals as well as blood pressure and flow status data were recorded before, during and after two consecutive 30-min EECP in 40 healthy adults (female/male: 17/23; age: 23.1 ± 2.3 years). HEP amplitude, frequency domain heart rate variability, electroencephalographic power and hemodynamic measurements of 21 subjects (female/male: 10/11; age: 22.7 ± 2.1 years) receiving active EECP were calculated and compared with those of 19 sham control subjects (female/male: 7/12; age: 23.6 ± 2.5 years).

**Results:** EECP intervention caused immediate obvious fluctuations of HEP from 100 to 400 ms after T-peak and increased HEP amplitudes in the (155–169) ms, (354–389) ms and (367–387) ms time windows after T-peak in the region of the frontal pole lobe. The modifications in HEP amplitude were not associated with changes in the analyzed significant physiological measurements and hemodynamic variables.

**Discussion:** Our study provides evidence that the HEP is modulated by immediate EECP stimuli. We speculate that the increased HEP induced by EECP may be a marker of enhanced brain-heart coupling. HEP may serve as a candidate biomarker for the effects and responsiveness to EECP.

## 1 Introduction

Enhanced external counterpulsation (EECP) is a safe, effective, and low-cost non-invasive assisted circulation technique, which is approved by the (Food and Drug Administration) FDA for the treatment of angina, acute myocardial infarction, congestive heart failure, as well as various ischemic cardio-cerebrovascular and neurological diseases. EECP employs electrocardiogram-gated sequential inflation of cuffs wrapped around calves, thighs and buttocks, which inflate and deflate during the cardiac cycle with certain pressure parameters, and has been shown to influence the cardiac and cerebral blood flow. EECP therapy also reduces myocardial ischemia symptoms, angina episodes and rehospitalization rates, and improves cardiac function, exercise tolerance, dysautonomia and quality of life (Shen et al., 2020). The immediate effects of EECP focusing on hemodynamics and vascular biology have been studied extensively (Tian et al., 2021; Zhang et al., 2021). Enhancement in diastolic aortic pressure, ventricular function, coronary and cerebral blood flow, reduction in left ventricular afterload, improvement in shear stress and endothelial function are postulated to be responsible for the aforementioned clinical benefits of EECP (Michaels et al., 2002; Bonetti et al., 2003; Liu et al., 2013; Raza et al., 2017; Shen et al., 2020). As the exploration of mechanisms deepens, the effects of EECP including inhibition of oxidative stress, anti-inflammation, vasculogenesis and angiogenesis have been recently revealed (Shen et al., 2020). Subsequently, studies of EECP have broadened in conditions like type 2 diabetes, erectile dysfunction, and psychiatric diseases (Springer et al., 2001; Raeissadat et al., 2018; Zeng et al., 2022). It is important to learn more about the effects of EECP and to explore potential mechanisms that will lead to optimized therapy and aid prognosis. However, within the field of EECP effects research, electroencephalography (EEG) and electrocardiography (ECG) signals processing have been seldom or separately studied so far. In addition, the acute brain-heart coupling responses to EECP are still obscure and need to be elucidated.

The brain-heart coupling is a bidirectional process that involves interactions between the central nervous system (CNS) and the autonomic nervous system (ANS) (Cortese et al., 2022). The brain regulates cardiac activity directly through the parasympathetic and sympathetic branches of the ANS, while the cardiovascular system feedback is transmitted by the vagus nerve *via* the activation of neuro-cardiac reflexes to influence brain functions (Silvani et al., 2016). The neuro-cardiac interaction, facilitated by the afferent and efferent vagus nerve of the ANS, is critical in maintaining homeostasis and adapting to environmental stimulations (Billman, 2020). Since the beneficial clinical effects of EECP may be related to changes in cardiac and/or cerebral activity, brain-heart coupling responses during or after EECP intervention might also play an indispensable role in relieving clinical symptoms. It is therefore important to establish a useful biomarker for exploring the potential mechanisms underpinning the acute neuro-cardiac effects of EECP.

Heartbeat evoked potential (HEP), which pertains to the cortical processing of afferent signals from the cardiovascular system, has been considered a neurophysiological indicator of interoception (Schulz et al., 2018). It is an EEG event-related potential obtained by averaging EEG signals time-locked to the R-peak or T-peak of simultaneous ECG recording (Kumral et al., 2022). The amplitude of HEP, linked to the phase of neural activity, reflects a synchronization or strength of brain-heart coupling based on afferent and efferent pathways (Tumati et al., 2021). Abundant recent evidence indicates that the brain-heart coupling quantified by HEP analysis is altered in different physiological or pathological conditions of emotional dysregulation including anxiety disorder, borderline personality disorder, depression and obsessive-compulsive disorder (Yoris et al., 2017; Schmitz et al., 2020; Tumati et al., 2021; Bogdány et al., 2022). The HEP technique has also been used to validate behavior measures or external interventions, including spontaneous breathing on cardiac interoception in healthy humans (Zaccaro et al., 2022), repetitive transcranial magnetic stimulation aimed at interoceptive signal processing (Mai et al., 2019), and transcutaneous auricular vagus nerve stimulation focusing on the activation of the interoceptive pathways (Poppa et al., 2022). Collectively, these findings indicate that HEP is associated with health status and is affected by stimuli or interventions. So HEP may provide clues for the brain-heart coupling effects of EECP.

Taken together, there is evidence of an association of EECP stimuli with the altered cardiovascular system, heart rate variability (HRV) as well as cerebral activity in both patients with various diseases and healthy subjects, but barely anything is known about the acute brain-heart coupling responses to EECP and its neural correlates. Moreover, the revelation of bidirectional neuro-cardiac interaction changes under the intervention of EECP might allow for the clarification of potential EECP mechanisms and optimization of the existing EECP technique. Consequently, to test the hypothesis that brain-heart coupling quantified by HEP using simultaneous EEG/ECG recordings is altered under EECP intervention, we assessed HEP parameters in healthy adults in a randomized, sham-controlled study. To answer the question of whether presumed alterations of HEP are related to parasympathetic modulation or cardiovascular changes, we additionally explored its association with physiological and hemodynamic measurements.

## 2 Materials and methods

### 2.1 Participants and study design

Healthy and physically active students from Beijing Sport University and Chinese PLA Medical School, performing moderate-intensity exercise 3–5 times per week, were included in the study between 8 June 2021, and 10 September 2021. Participants were eligible if they were 18–30 years old, right-handed, free of past and present medical conditions including psychiatric, neurological, or cardiological disease, non-user of nicotine, caffeine, or alcohol products, and not taking medication that could affect the ANS (all by self-report). Cardiovascular status (diagnosed by GE Marquette Mac 5000 EKG system), hemodynamic characteristics (assessed by CNAP Monitor 500), the clinical hematological and biochemical tests (performed by Sysmex 9,100 automated machine and Roche Cobas 8,000 system based on venous blood samples) of all the participants were screened and checked. The study was approved by the Institutional Review Committee of the Chinese PLA General Hospital. These trials complied with the Declaration of Helsinki and all subjects gave informed consent in written form. In addition, the study was pre-registered at the Chinese Clinical Trial Registry (http://www.chictr.org.cn; ChiCTR2000033645; 06/07/2020).

**FIGURE 1:**
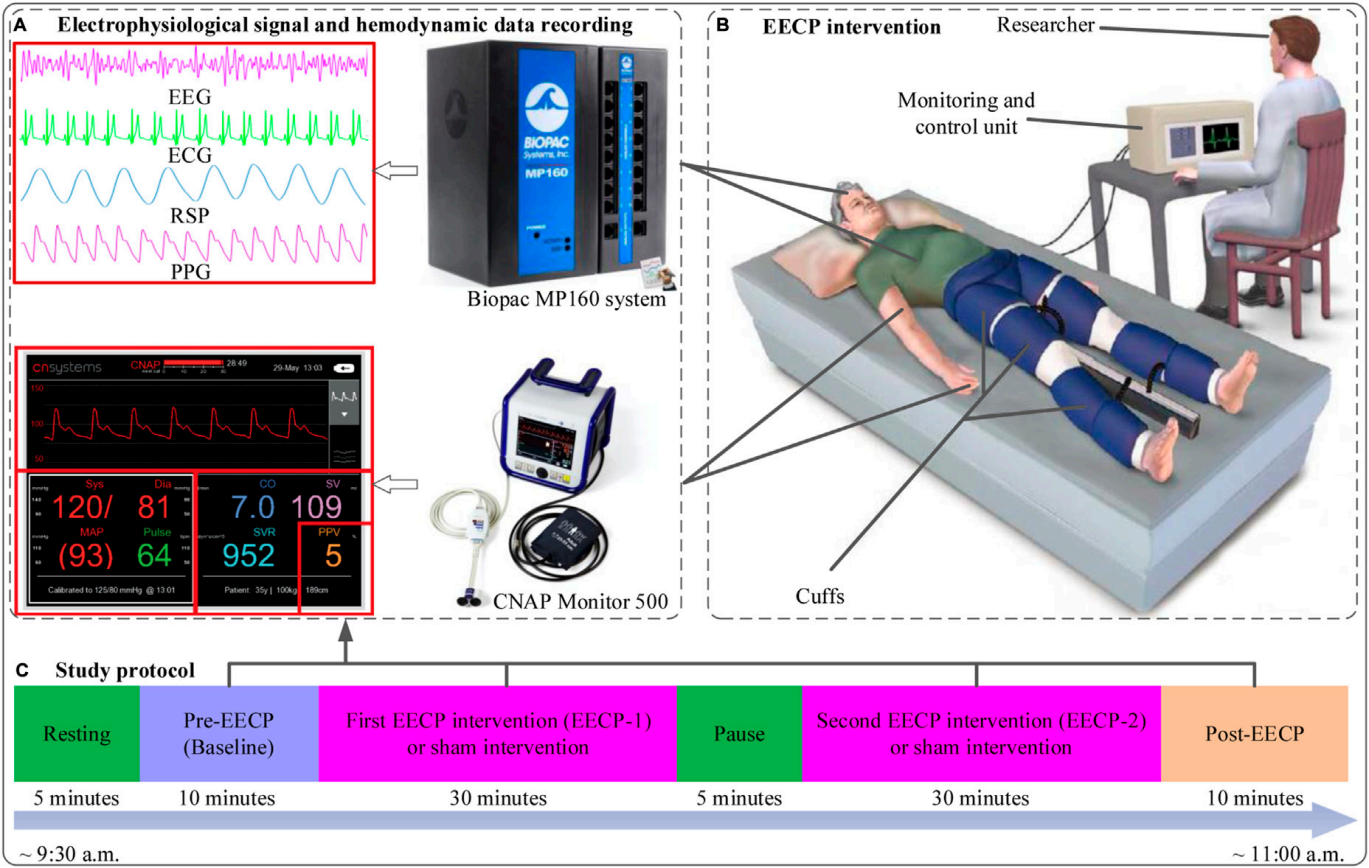
The study protocol, flow chart of enhanced external counterpulsation (EECP) intervention, physiological signal and hemodynamic data acquisition **(A)** Electrophysiological signals (EEG, ECG, RSP and PPG) and hemodynamic data (SBP, DBP, MAP, CO, CI, SVV and SVR) recording **(B)** EECP intervention in sham (with pressure set at 0 MPa) and active (with pressure set at 0.020 MPa) groups (revised based on the figure from https://edtreatment.info/enhanced-external-counterpulsation-eecp-therapy-for-erectile-dysfunction/) **(C)** Study protocol: about 90 min including resting (5 min), Pre-EECP (Baseline for 10 min), first EECP intervention (EECP-1 for 30 min), a pause of 5 min, second EECP intervention (EECP-2 for 30 min) and Post-EECP (10 min) stages.

Participants were randomly assigned (1:1) to either intervention with active EECP stimulation (EECP group) or intervention with sham EECP stimulation (control group receiving EECP with 0 MPa pressure) using a simple computer-generated sequence. As shown in Figure 1, the protocol study lasted about 90 min and consisted of stages including resting, pre-EECP (Baseline), first EECP intervention (EECP-1), a pause of 5 min, second EECP intervention (EECP-2) and Post-EECP. In the resting stage, participants lay on the EECP treatment bed for 5 min of relaxation in a supine position with their legs and buttocks wrapped in cuffs to eliminate the impact of physical and mental activity on the physiological signal. The resting stage is followed by a 10-min period we called pre-EECP for control purposes. Afterward, two sessions of 30-min active EECP stimulation or sham EECP stimulation were administered, with a pause of 5 min between the two sessions of EECP intervention for recovery and to keep the participant awake. Finally, the 10 min recovery period immediately after the EECP-2 was defined as the Post-EECP stage, in which the EECP system was turned off. Physiological signal and hemodynamic data during the Baseline, EECP-1, EECP-2 and Post-EECP periods were recorded in the morning hours (9:35–11:00 a.m.) in a quiet EECP treatment chamber with the temperature controlled at (22–25) °C. During each data recording session, subjects were asked to be in a resting state with a supine position. Forty-six participants were recruited, but three were later dropped due to the discovery of blood biochemical test abnormalities. Hence, 43 subjects participated in the EECP study. However, 40 participants (21 active EECP intervention, female/male: 10/11, age: 22.7 ± 2.1 years; 19 sham control, female/male: 7/12; age: 23.6 ± 2.5 years) were included in the final analyses as EEG recordings of three participants were lost due to a loose connection in the electrodes.

### 2.2 Enhanced external counterpulsation

The EECP system (P-ECP/TI, Chongqing PSK-health Sci-Tech Development Co., Ltd, China) consists of an ECG and photoplethysmography (PPG) monitor, a console for parameter setting, two sets of three cuffs, an air compressor and a treatment bed. Before an EECP stimuli session, cuffs are wrapped around the patient’s calves, thighs and buttocks. At the beginning of diastole, pressure is rapidly applied *via* cuffs in sequence synchronized with the cardiac cycle from the lower calves to the upper thighs. This process increases arterial blood pressure, blood flow and venous return. At the end of diastole, pressure is deflated instantaneously from all the cuffs before the onset of systole, allowing the compressed vessels to reconfirm, thereby reducing vascular impedance (Arora et al., 1999). In the present study, the EECP intervention pressure was set at 0.020 Mpa in the active EECP group and 0 Mpa in the sham control group under the operation of an experienced and trained physician. All other conditions of intervention delivery are the same in both groups.

### 2.3 Data acquisition

Single-channel EEG, ECG, PPG, and thoracic bio-electrical impedance-based respiration (RSP) were acquired simultaneously by a Biopac MP160 system (Biopac System Inc Goleta, CA, United States). The unipolar EEG signal was taken on the Fpz position, which is a characteristic electrode of the frontal pole lobe according to the international 10–20 EE G system. The reference and ground electrodes were placed in A1, the mastoid bone close to the ear. The ECG signal was acquired using lead II electrode placement—one below the right mid-clavicle and the other on a lower left rib, with a ground electrode placed on the lower right rib. Physiological signals were sampled at 2000 Hz and exported in MAT format. Continuous hemodynamic variables include systolic blood pressure (SBP), diastolic blood pressure (DBP), mean arterial pressure (MAP), cardiac output (CO), cardiac index (CI), stroke volume variability (SVV), systemic vascular resistance (SVR), as well as arterial blood pressure (AP) were recorded non-invasively at a sampling frequency of 100 Hz using a CNAP Monitor 500 (CNSystems Medizintechnik, Graz, Austria). The CNAP double finger cuff was placed on the index and middle finger of the left arm, while the upper arm cuff was attached to the ipsilateral arm. Hemodynamic data were output in TXT format. All acquired data were stored on a personal computer for further offline analysis.

### 2.4 Signal preprocessing

The physiological signals were preprocessed by Acqknowledge software version 5.0 (Biopac System Inc Goleta, CA, United States) and MATLAB R2020 (MathWorks, Natick, MA, United States). Continuous raw EEG data were filtered with an (0.05–100) Hz bandpass filter. A notch filter at 50 Hz was applied to remove power line interference, and artifact rejection was performed by visual inspection to remove the non-physiological artifacts. The EEG signal was then analyzed by discrete wavelet with Daubechies db4 to obtain approximate and detail components, which were processed by complete empirical mode decomposition for adaptive noise to calculate the intrinsic mode functions. Thereafter, independent component analysis was used to compute the independent components of intrinsic mode functions followed by sample entropy quantification. The independent component corresponding to the sample entropy values satisfying the Gomez Herrero condition was regarded as an artifact and set to zero (Gomez Herrero et al., 2006). Finally, the inverse independent component analysis was then performed to reconstruct the new approximate and detail components, based on which the EEG signal with the rejection of cardiac field artifact, eye movement artifact, and muscle artifacts was obtained (Hu et al., 2022) (Supplementary Figures S1–S6) R-peak and T-peak were detected and labeled automatically with the help of peak detector functions integrated into Acqknowledge and Kubios HRV (Kubios 3.4, University of Eastern Finland, Kuopio) software. The interbeat interval (IBI) between 300 and 2000 ms, consecutive IBI differences ≤200 ms, and prolongations or shortenings ≤20% than the average of five preceding sinus rhythm IBIs were considered as sinus rhythm QRS complexes (Liu et al., 2020). Thereafter, automatically annotated results were carefully visually inspected and manually corrected by editing ectopic beats, arrhythmias and noise to suppress computational errors. The continuous hemodynamic signals for Baseline, EECP-1, EECP-2 and Post-EECP stages were preprocessed by 50 Hz notch filtering, and then the mean value of each hemodynamic signal for the corresponding stage was calculated. For Baseline and Post-EECP stages, 10 min of physiological and hemodynamic data were used for feature extraction. While for EECP-1 and EECP-2 stages, the 30-min physiological and hemodynamic data were divided into three segments on an average of 10 min, the parameters of each segment were extracted and averaged for further analysis.

### 2.5 Heartbeat evoked potential

**FIGURE 2:**
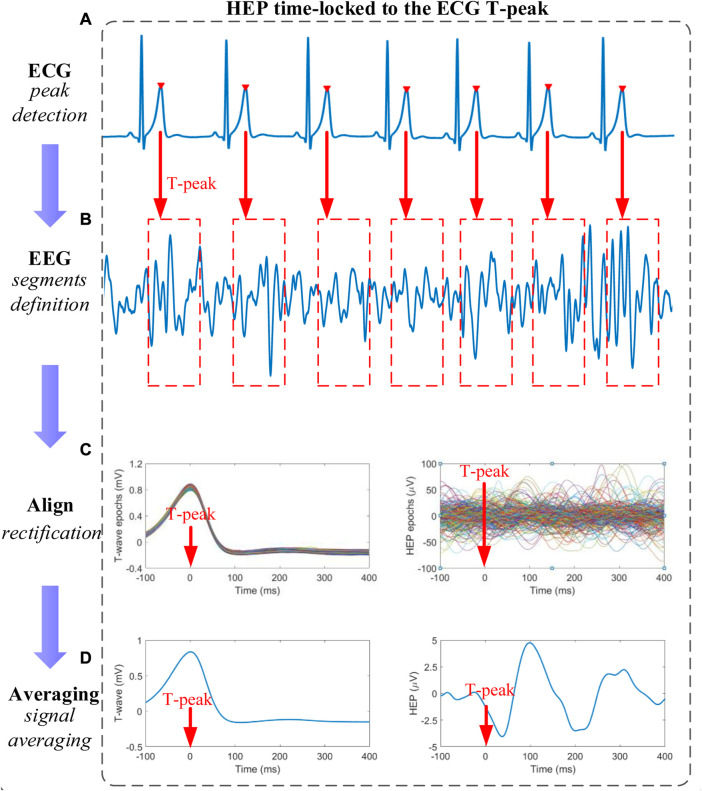
Schematic representation of heartbeat evoked potential (HEP) analysis **(A)** T-peaks detection of electrocardiography (ECG) signals **(B)** Definition of time-locked T-peaks electroencephalography (EEG) segments with a certain length **(C)** All EEG segments were aligned relative to the T-peak in segments after baseline correction and outliers-exclusion **(D)** The HEP curve is obtained by averaging the signals within the aligned segments.

The ECG T-peak event, which approximately corresponds to each EECP inflation time (EECP onset) in the present study was selected as a temporal reference. After preprocessing, resulting continuous EEG data were segmented relatively to the detected T peaks of simultaneous ECG signal in epochs ranging from 100 ms before the T peaks to 400 ms after the T peaks. Segments of T-peak-triggered EEG were aligned and averaged for the computation of HEP. Based on the MATLAB software, all segments were manually reviewed for artifact identification and sweeps with EEG activity above 100 μV were excluded from further analysis (Zaccaro et al., 2022). In addition, a baseline correction was also performed by subtracting the mean of the first 100 ms before the T peak from the entire HEP average for each individual (Zaccaro et al., 2022). The time window of interest for the statistical analysis was set to (80–400) ms after T-peak, coincident with cardiac relaxation when the cardiac field artifact is minimum (Dirlich et al., 1997; Immanuel et al., 2014). Finally, the mean amplitude of HEP within the time window of interest after the T peak was determined (Figure 2).

### 2.6 ECG and EEG power spectral density analysis

Epochs of IBI time series of 10-min length were interpolated (equidistant sampling at 4 Hz) and detrended for spectral HRV analysis based on the fast Fourier transform. The frequency domain measures were computed from the power spectral density estimate for each frequency band including absolute power values of low frequency (LF, 0.04–0.15 Hz), high frequency (HF, 0.15–0.40 Hz) and LF/HF power ratio (Task Force of the European Society of Cardiology and the North American Society of Pacing and Electrophysiology, 1996).

The resulting EEG signals were filtered into four different frequency bands: Delta band (*δ*, 0.5–4 Hz), Theta band (*θ*, 4–7 Hz), Alpha band (*α*, 8–13 Hz), and Beta band (*β*, 14–30 Hz) (Cox and Fell, 2020). Welch’s periodogram method was used to estimate the power spectral density with non-overlapping Hanning windows of 4 s for each 10-min EEG segment. The power spectral density of the *δ*, *θ*, *α*, *β* and band power ratios *α/β* were calculated by an average computation.

### 2.7 Hemodynamic data analysis

The collected hemodynamic data were segmented according to the stages before, during (two 30-min EECP interventions), and after the EECP interventions. The average values for each segment were calculated to obtain hemodynamic parameters including systolic blood pressure (SBP, mmHg), diastolic blood pressure (DBP, mmHg), mean arterial pressure (MAP, mmHg), cardiac output (CO, L/min), cardiac index (CI, L/min m^2^), stroke volume variability (SVV, %), and systemic vascular resistance (SVR, dyns/cm^5^).

### 2.8 Correlation analyses with HEP

To test whether potential HEP differences were associated with changes in the analyzed physiological measurements during EECP, exploratory correlation analyses were performed between ΔHEP (ΔHEP are changes in HEP amplitude of significant epochs from baseline to EECP-1 and EECP-2 stages) and variation of possible significant characteristic parameters of ECG, EEG as well as hemodynamics. More precisely, Spearman correlations between the mean ΔHEP amplitudes of the significant HEP epochs after the T-peak and variations of physiological indices were computed for stages EECP-1 and EECP-2, respectively. The results were adjusted using the false discovery rate method.

### 2.9 Statistical analysis

Statistical analyses were performed using the SPSS version 20 software package (SPSS, Chicago, Ill, United States). Kolmogorov-Smirnov tests were applied to determine the normal distribution of sample data. The difference in continuous sample characteristics between the active EECP and sham control groups was tested by the Mann-Whitney U test, while the qualitative or categorical variables were analyzed by Fisher’s exact tests. A non-parametric cluster-based permutation test (Maris and Oostenveld, 2007), implemented in MATLAB script (Krol et al., 2018), was applied to compare within-group HEP differences under certain conditions (EECP-1 vs. Baseline, EECP-2 vs. Baseline, and Post-EECP vs. Baseline). To determine the distribution of maximal cluster-level statistics obtained by chance, condition labels were randomly shuffled 1,000 times. HEP samples within the time window (80–400) ms after T-peak with t values exceeding the threshold (cluster threshold *p*-value is set at 0.05) became cluster candidates and were subsequently clustered based on their temporal adjacency, then the cluster statistics are quantified by taking the sum of t values of all points within each cluster (Al et al., 2020). The dependent variables including the mean amplitude of significant HEP clusters, HRV measurements, EEG band powers and hemodynamic parameters in Baseline, EECP-1, EECP-2, and Post-EECP periods as well as between the active EECP group and sham control group were tested by two-way repeated measures ANOVA (intervention factor: EECP vs. Sham; intervention phase factor: Baseline, EECP-1, EECP-2 and Post-EECP) with Bonferroni *post hoc* analysis. The effect size of each factor was computed as partial eta-squared (*η*_p_^2^): a value of *η*_p_^2^ of 0.010 was considered a small effect, a value of 0.059 a medium effect, and a value of 0.138 a large effect (Carta et al., 2021). A value of *p* < 0.05 was considered to indicate statistical significance.

## 3 Results

No adverse events or obvious protocol deviations affecting the safety, wellbeing or rights of participants or the scientific integrity of the study were reported. There were no significant differences in baseline sample characteristics between the active EECP group and the sham control group. Details about demographic and clinical status are shown in Table 1.

**TABLE 1:** Baseline sample characteristics.

	EECP group (*n* = 21)	Control group (*n* = 19)	*p*-value
Age (years)	22.7 ± 2.1	23.6 ± 2.5	0.169
Female/male	10/11	7/12	0.538
BMI (kg·m^-2^)	21.5 ± 2.7	22.6 ± 2.2	0.244
SBP (mmHg)	105 ± 16	110 ± 10	0.284
DBP (mmHg)	68 ± 14	64 ± 8	0.154
MAP (mmHg)	83 ± 12	82 ± 8	0.431
Heart rate (bpm)	65 ± 11	67 ± 10	0.316
Respiratory rate (rpm)	18 ± 2	19 ± 3	0.632
SpO_2_ (%)	97 ± 1	96 ± 2	0.080
Hemoglobin (g·L^-1^)	138 ± 13	144 ± 13	0.212
Red blood cell (10^12^ L^-1^)	4.63 ± 0.44	4.71 ± 0.53	0.745
White blood cell (10^9^ L^-1^)	6.69 ± 1.39	6.09 ± 1.37	0.239
Platelets (10^9^ L^-1^)	224 ± 60	237 ± 54	0.542
Glucose (mmol·L^-1^)	5.37 ± 0.89	5.12 ± 0.71	0.694
Uric acid (µmol·L^-1^)	327.4 ± 75.6	344.0 ± 94.2	0.828
Cholesterol (µmol·L^-1^)	4.15 ± 0.75	3.92 ± 0.66	0.424
Creatine kinase (mmol·L^-1^)	122.2 ± 80.6	139.9 ± 116.1	0.310
Potassium (mmol·L^-1^)	4.12 ± 0.19	4.11 ± 0.28	0.924
Cystatin C (mg·L^-1^)	0.77 ± 0.10	0.73 ± 0.13	0.284

Values are mean ± SD, or number unless otherwise indicated. *p*-values for group comparisons were tested using Mann-Whitney U tests or Fisher’s exact tests where appropriate. BMI, body mass index; SBP, systolic blood pressure; DBP, diastolic blood pressure; MAP, mean arterial pressure; SpO_2_, saturation of peripheral oxygen.

**FIGURE 3:**
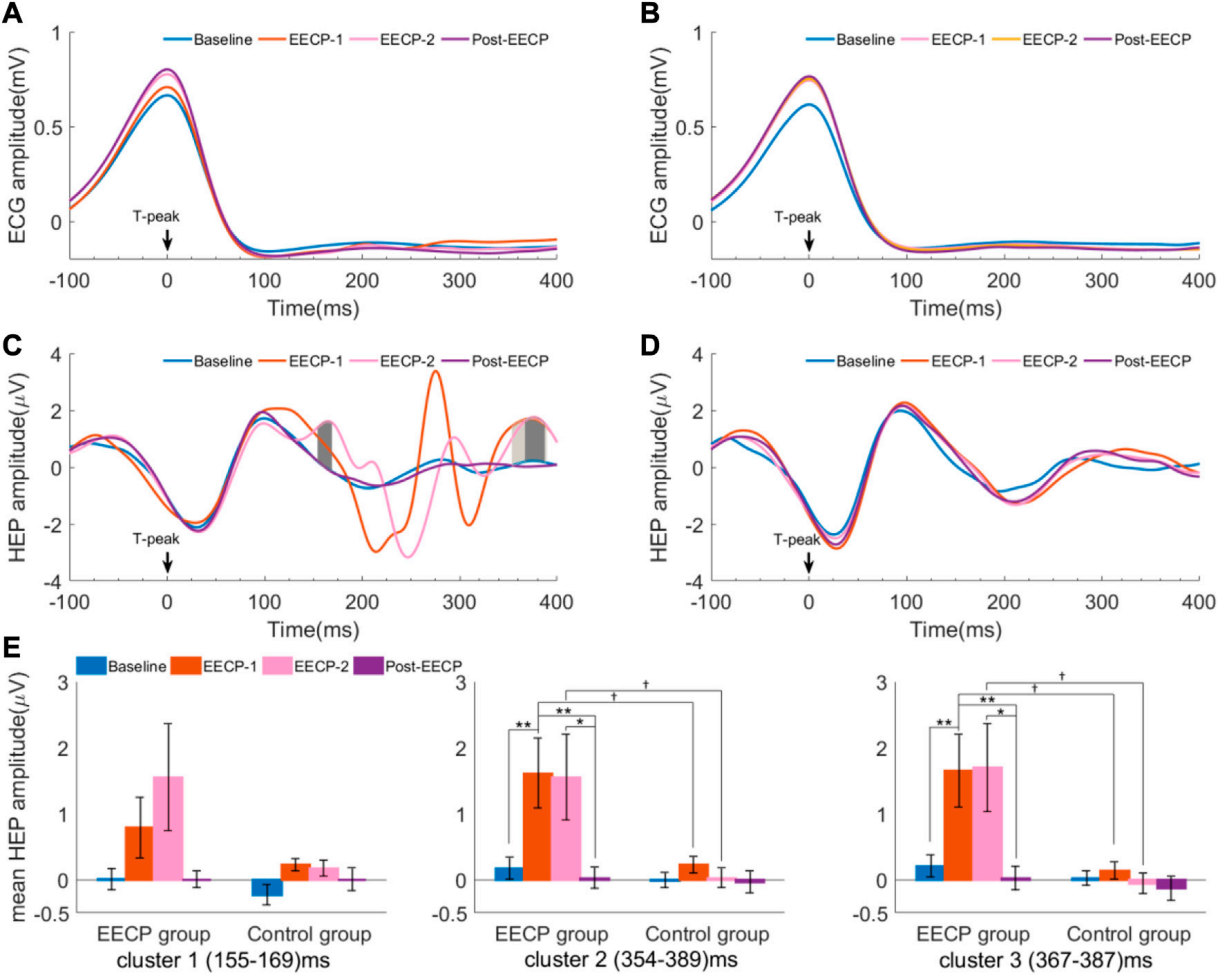
Heartbeat evoked potential (HEP) differences in Baseline, EECP-1, EECP-2, and Post-EECP periods for both active EECP and sham control groups **(A)** and **(B)** are ECG amplitude relative to T-peaks for the active EECP group and sham control group, respectively **(C)** Three positive clusters with significant differences in the time windows (155–169) ms (deep gray, EECP-2 vs. Baseline), (354–389) ms (gray, EECP-1 vs. Baseline), and (367–387) ms (deep gray, EECP-2 vs. Baseline) after T-peak were observed in the active EECP group (cluster-based permutation test) **(D)** No significant differences were found in the HEP amplitudes for the sham control group among 4 periods **(E)** Mean HEP amplitude (values are mean ± SEM) of significant clusters for active EECP and sham control groups before, during, and after two sessions of 30 minute-EECP. HEP differences were estimated using cluster-based permutation test. *p*-values for HEP cluster comparisons were tested by two-way repeated measures ANOVA with Bonferroni *post hoc* analysis. ^†^ *p* < 0.05 (active EECP group compared to sham EECP group); ^*^ *p* < 0.05, ^**^ *p* < 0.01 (comparison among EECP intervention phases in active EECP group).

HEP analysis for both groups in Baseline, EECP-1, EECP-2, and Post-EECP periods are depicted in Figure 3. In active EECP individuals, the neural responses time-locked to the T-peak showed obvious fluctuations during two sessions of EECP intervention (Figure 3C), whereas the HEPs in the same periods were more flattened in sham control subjects (Figure 3D). In addition, we assessed HEPs changes during Baseline, EECP-1, EECP-2 and Post-EECP in the time window of 0 (EECP intervention onset) to 400 ms after T-peak with a cluster-based permutation test. We found 3 significant positive clusters during EECP intervention in the active EECP group: 155 ms–169 ms (cluster 1 during EECP-2, t_28_ = −0.244, *p* = 0.001, Cohen’s d = 0.675), 354 ms–389 ms (cluster 2 during EECP-1, t_70_ = −0.470, *p* = 0.001, Cohen’s d = 0.763), and 367 ms–387 ms (cluster 3 during EECP-2, t_40_ = 0.580, *p* = 0.001, Cohen’s d = 0.675) after T-peak. However, HEPs for the sham control group before, during and after EECP did not differ significantly. The results indicate that EECP increases the HEP amplitude in certain time windows after T-peak (EECP onset).

Moreover, we used a two-way repeated measures ANOVA with the factors intervention (EECP vs. sham) and intervention phase (Baseline, EECP-1, EECP-2 and Post-EECP) to examine their effect on the mean amplitude of HEP for significant clusters (Supplementary Table S1). When considering the main effects of EECP, the mean HEP amplitude of cluster 2 (EECP-1: 1.61 ± 0.53 vs. 0.23 ± 0.12; EECP-2: 1.56 ± 0.65 vs. 0.03 ± 0.15, F_1,38_ = 11.311, *p* = 0.002, *η*_p_^2^ = 0.229) and cluster 3 (EECP-1: 1.65 ± 0.55 vs. 0.14 ± 0.13; EECP-2: 1.70 ± 0.67 vs. −0.05 ± 0.16, F_1,38_ = 11.244, *p* = 0.002, *η*_p_^2^ = 0.228) between active EECP and sham EECP groups exhibit significant differences (Figure 3E). The subjects that received active EECP intervention showed significant changes in the mean HEP amplitude of cluster 2 (Baseline: 0.18 ± 0.17; EECP-1: 1.61 ± 0.53; EECP-2: 1.56 ± 0.65; Post-EECP: 0.03 ± 0.16, F_3,38_ = 3.966, *p* = 0.022, *η*_p_^2^ = 0.094) and cluster 3 (Baseline: 0.21 ± 0.17; EECP-1: 1.65 ± 0.55; EECP-2: 1.70 ± 0.67; Post-EECP: 0.03 ± 0.18, F_3,38_ = 4.114, *p* = 0.017, *η*_p_^2^ = 0.098) in different intervention phases, while the sham control group did not demonstrate this effect. The results indicated that HEP was affected by the active EECP and sham EECP conditions.

**FIGURE 4:**
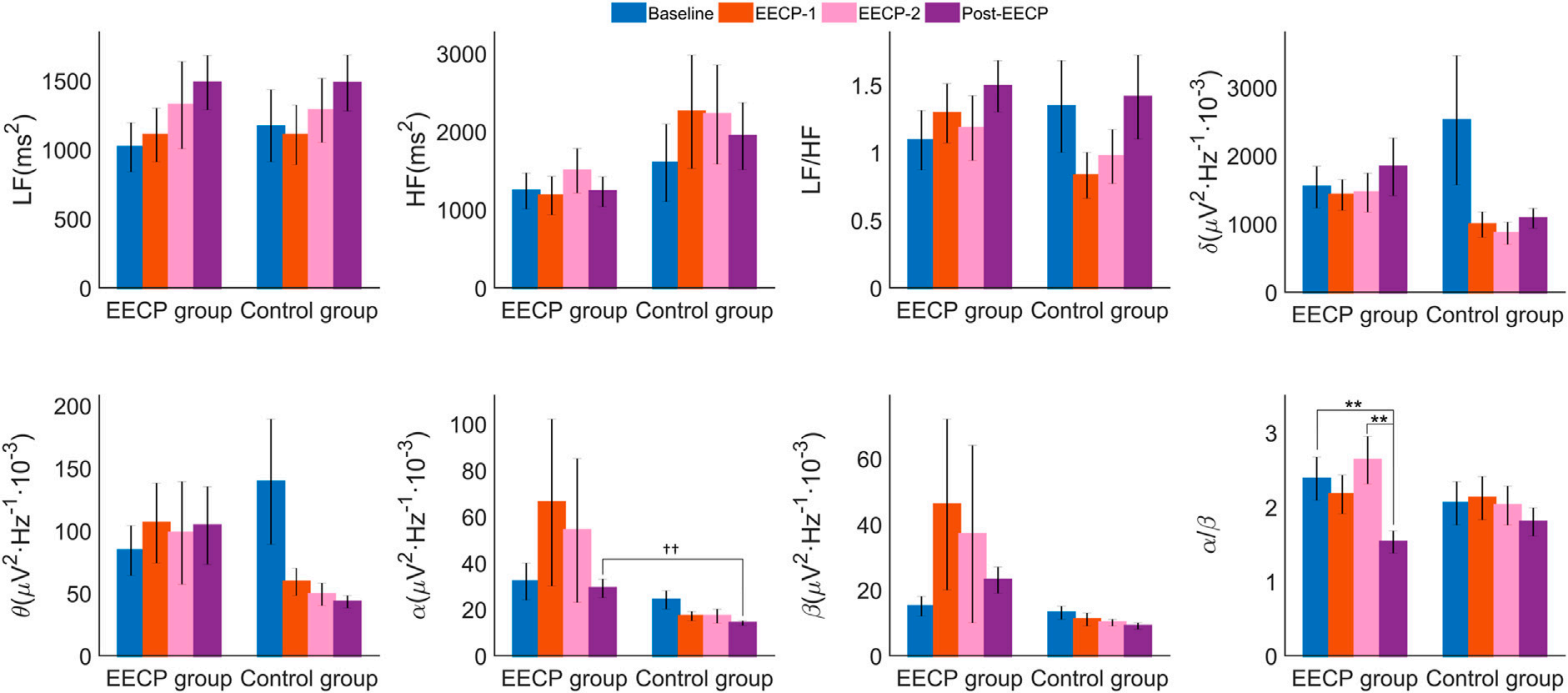
Power spectral density (values are mean ± SEM) of heart rate variability (HRV) and electroencephalography (EEG) in active EECP and sham control groups before, during, and after two sessions of 30 minute-EECP. LF, low frequency (HRV frequency band 0.04–0.15 Hz); HF, high frequency (HRV frequency band 0.15–0.40 Hz); δ, Delta frequency band of EEG (0.5–4) Hz; θ, Theta frequency band of EEG (4–7) Hz; α, Alpha frequency band of EEG (8–13) Hz; β, Beta frequency band of EEG (14–30) Hz. *p*-values for within-group or inter-group comparisons were tested by two-way repeated measures ANOVA with Bonferroni *post hoc* analysis. ^††^ *p* < 0.01 (active EECP group compared to sham EECP group); ^**^ *p* < 0.01 (comparison among EECP intervention phases in active EECP group).

As shown in Figure 4 and Supplementary Table S2, no main effects of intervention, intervention phase, or interaction effects were observed for the analyzed frequency-domain measures of HRV including LF, HF and LF/HF (all *p* > 0.05). However, a significant main effect of the intervention (F_1,38_ = 4.611, *p* = 0.038, *η*_p_^2^ = 0.108) was observed for α frequency band power spectral density of EEG. The α power spectral density of the Post-EECP phase (14 ± 1 vs. 29 ± 4, *p* = 0.004) in the sham control group was significantly lower than that of the active EECP group. In addition, a significant main effect of the intervention phase (F_3,38_ = 4.547, *p* = 0.009, *η*_p_^2^ = 0.107) was also observed for α/β, such that two sessions of EECP reduced α/β in the Post-EECP phase compared to those of stages Baseline (1.53 ± 0.15 vs. 2.38 ± 0.29, *p* = 0.002) and EECP-2 (1.53 ± 0.15 vs. 2.63 ± 0.32, *p* = 0.002) in the active EECP group.

**FIGURE 5:**
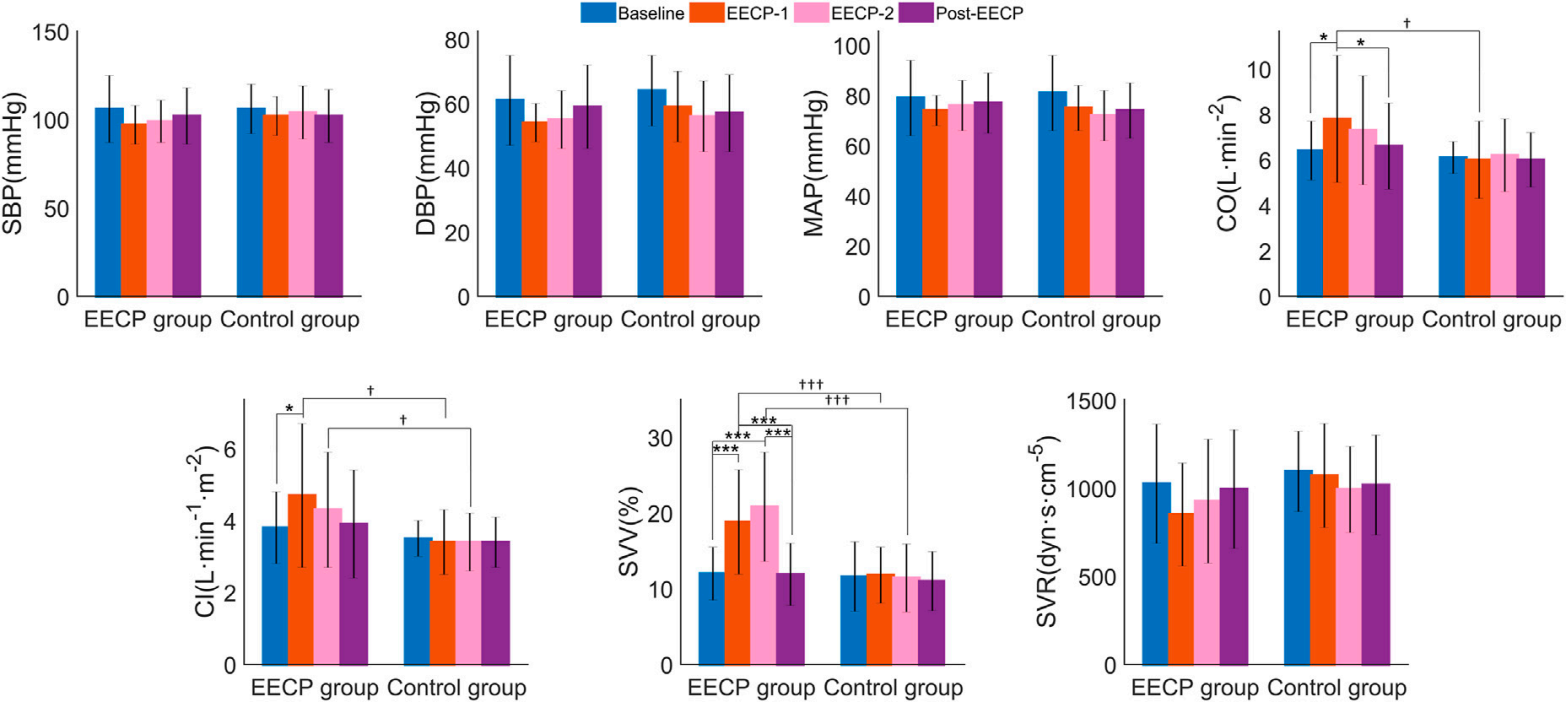
The hemodynamic parameters (values are mean ± SD) of subjects in active EECP and sham control groups before, during, and after two sessions of 30 minute-EECP. SBP, systolic blood pressure; DBP, diastolic blood pressure; MAP, mean arterial pressure; CO, cardiac output; CI, cardiac index; SVV, stroke volume variability; SVR, systemic vascular resistance. ^†^ *p* < 0.05, ^†††^ *p* < 0.001 (active EECP group compared to sham EECP group); *p*-values for within-group or inter-group comparisons were tested by two-way repeated measures ANOVA with Bonferroni *post hoc* analysis. ^*^ *p* < 0.05, ^***^ *p* < 0.001 (comparison among EECP intervention phases in active EECP group).

Two-way ANOVA (Supplementary Table S3) revealed significant effects of factor intervention on CO (F_1,38_ = 4.168, *p* = 0.048, *η*_p_^2^ = 0.099), CI (F_1,38_ = 4.949, *p* = 0.032, *η*_p_^2^ = 0.115) and SVV (F_1,38_ = 14.191, *p* = 0.001, *η*_p_^2^ = 0.272), significant effects of factor intervention phase on DBP (F_3,38_ = 5.381, *p* = 0.003, *η*_p_^2^ = 0.124), MAP (F_3,38_ = 3.827, *p* = 0.016, *η*_p_^2^ = 0.092) and SVV (F_3,38_ = 14.598, *p* < 0.001, effect size = 0.278), and interaction effects on CI (F_3,38_ = 3.085, *p* = 0.038, *η*_p_^2^ = 0.075) and SVV (F_3,38_ = 12.726, *p* < 0.001, *η*_p_^2^ = 0.251). As shown in Figure 5, *post hoc* analysis showed that CO for stages of EECP-1 (7.8 ± 2.8 vs 6.4 ± 1.3, *p* = 0.014), CI for stages of EECP-1 (4.7 ± 2.0 vs 3.8 ± 1.0, *p* = 0.014), and SVV for both stages of EECP-1 (18.8 ± 6.9 vs 12.0 ± 3.5, *p* < 0.001) and EECP-2 (20.8 ± 7.2 vs 12.0 ± 3.5, *p* < 0.001) were significantly increased compared to those of Baseline in the active EECP group. No significant changes in hemodynamic parameters were found in the sham control group. In addition, CO of stages EECP-1 (6.0 ± 1.7 vs 7.8 ± 2.8, *p* = 0.018), CI of stages EECP-1 (3.4 ± 0.9 vs 4.7 ± 2.0, *p* = 0.014) and EECP-2 (3.4 ± 0.8 vs 4.3 ± 1.6, *p* = 0.014), and SVV of stages EECP-1 (11.8 ± 3.7 vs 18.8 ± 6.9, *p* < 0.001) and EECP-2 (11.4 ± 4.5 vs 20.8 ± 7.2, *p* < 0.001) for the control group were significantly lower than those of corresponding stages for the EECP group.

**FIGURE 6:**
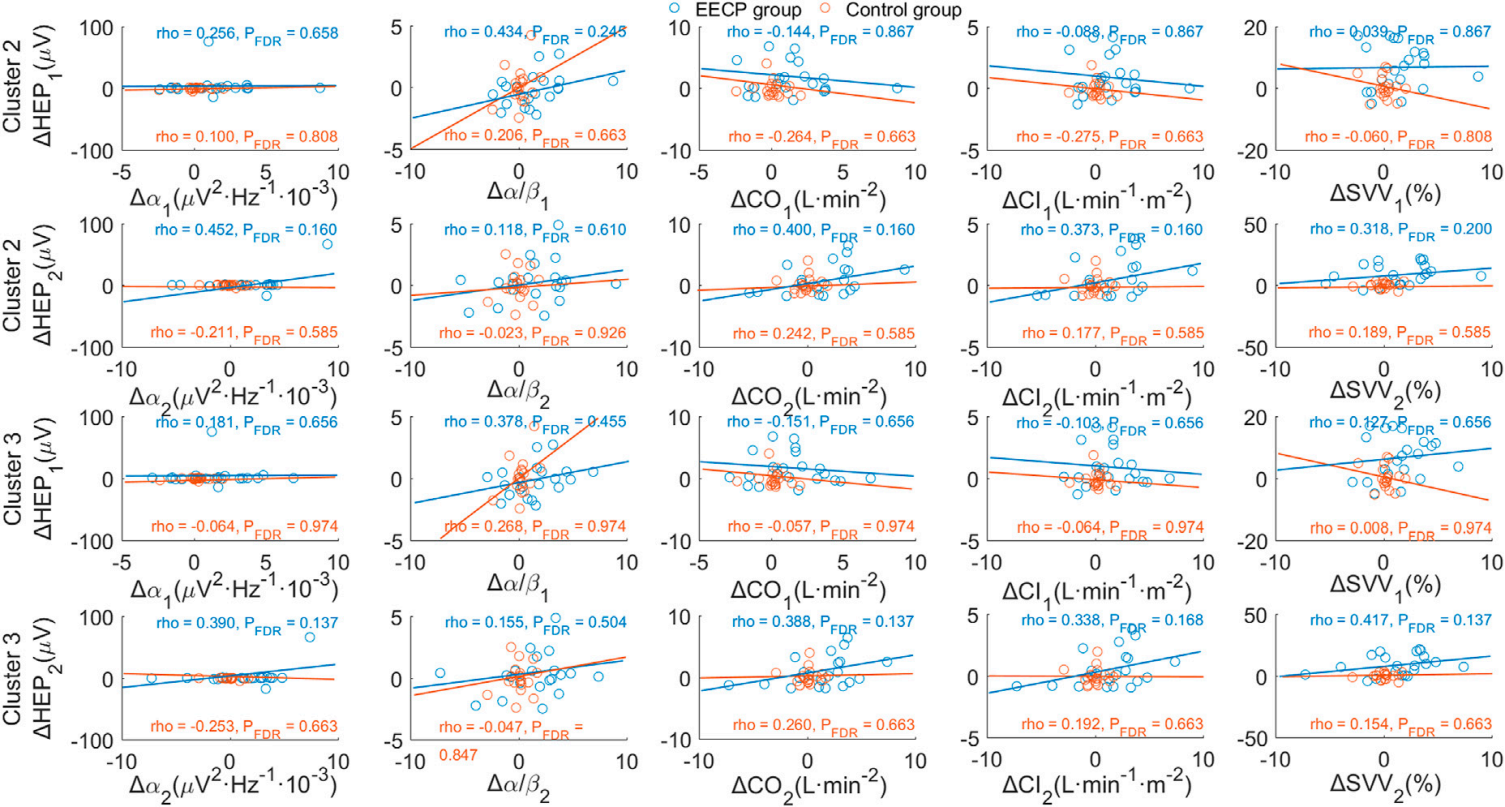
Correlation analysis of changes in HEP amplitude and variations of physiological parameters. *p*-values were calculated for Spearman’s correlation. The upper two panels show the association between ΔHEP of cluster 2 (354 ms–389 ms after T-peak) and variations of significant physiological parameters for stages EECP-1 and EECP-2, respectively. Similarly, the lower two panels demonstrate the association between ΔHEP of cluster 3 (367 ms–387 ms after T-peak) and variations of significant physiological parameters for stages EECP-1 and EECP-2, respectively. α, Alpha frequency band of EEG (8–13) Hz; α/β, power ratio of Alpha and Beta frequency bands; CO, cardiac output; CI, cardiac index; SVV, stroke volume variability.

The relationship between HEP amplitude changes from baseline (ΔHEP for cluster 2 and cluster 3) in stages EECP-1 and EECP-2 and corresponding variations of physiological parameters (Δα, Δα/β, ΔCO, ΔCI, and ΔSVV) with significance were analyzed through Spearman correlation. As shown in Figure 6 and Supplementary Table S4, results did not reveal any significant association between changes in mean HEP amplitude and variations of physiological measurements or hemodynamic parameters, neither in the active EECP group nor in sham control subjects (all P_FDR_ > 0.05).

## 4 Discussion

To the best of our knowledge, the present study is the first to investigate the acute effects of EECP on neural responses to cardiac signals with HEP analysis in healthy adults. Our results demonstrated that EECP modified HEP morphology and amplitude at the scalp of a certain region (Fpz) in the frontal lobe. Specifically, EECP produced obvious fluctuations of HEP from 100 to 400 ms after T-peak and increased HEP amplitudes in the (155–169) ms, (354–389) ms and (367–387) ms time windows after T-peak in the region of the frontal pole lobe electrode. We further revealed that EECP reduced band power ratios α/β of EEG and confirmed the immediate effects of EECP on hemodynamics. However, no significant correlations were observed between changes in HEP amplitude and variations of physiological measurements with significance. Taken together, our findings provide evidence for EECP’s immediate ability to induce the reorganization of cardiac-influenced neuronal activity and to enhance brain-heart coupling.

The characteristic of HEP modulated by EECP in the present study is consistent with previous studies, that is frontal lobe susceptibility in the EEG to pathophysiological change or active intervention related to central or autonomic function. Early topographical analysis of the HEP showed that the effects of attention on the amplitude of cortical response to heartbeat were more pronounced in frontal and central regions (Montoya et al., 1993). The study which addressed the association between HEPs and survival after cardiac arrest indicated that HEP amplitudes at frontopolar and frontal electrodes were negatively related to mortality (Schulz et al., 2018). In nightmare sufferers or patients with atrial fibrillation, significant negative clusters of HEP over the frontal lobe channels were observed with lower amplitudes in groups with patients compared to those of healthy controls (Perogamvros et al., 2019; Kumral et al., 2022). Since HEP is usually regarded as an objective electrophysiological biomarker of brain-heart interactions (Park & Blanke, 2019; Coll et al., 2021), the reduction of HEP amplitude suggests an attenuation of brain-heart coupling due to the pathological state. In addition, active interventions such as meditation and transcutaneous auricular vagus nerve stimulation were also found to produce lower amplitude HEPs in frontal sensors (Jiang et al., 2020; Poppa et al., 2022). From the neuroanatomical point of view, certain areas of the frontal lobe activated by EECP receive visceral projections through visceral afferent pathways, which contribute to bidirectional brain-heart interaction and dynamical regulation of homeostasis.

We observed a strong response of HEP morphology and amplitude to EECP for the difference between active intervention and sham. One possible confounding factor might be rhythmic noise introduced in the physiological signal acquisition electrodes when the EECP equipment operating. Since the EECP device in the sham control group also works normally under the triggering of ECG T wave during the process of EECP-1 and EECP-2 (the intervention pressure is 0 MPa), HEP did not show changes in morphology and amplitude. Therefore, the aforementioned confounder can be excluded and the determinant of our findings about HEP response to EECP stimuli might be something else. A potential explanation may be altered HEP due to changes in cardiac autonomic function or EEG characteristics. However, consistent with previous studies (Michaels et al., 2007; Akbarzadeh et al., 2011), we found no significant changes in vagal modulation and sympathovagal balance reflected by HF and LF/HF of HRV, respectively. A second possible explanation might be EECP-associated hemodynamic changes that lead to the alteration of neural responses. Although the changes in HEP did not relate to variations of CO, CI and SVV, we cannot completely exclude this explanation. Previous studies have shown that EECP has complex immediate hemodynamic effects, resulting in increased aortic diastolic pressure, decreased systolic pressure, increased cardiac output, and increased coronary and cerebral blood flow (Michaels et al., 2002; Liu et al., 2013; Raza et al., 2017; Shen et al., 2020). The redistribution of the blood flow, especially the cerebral blood flow and its changes induced by EECP that are closely related to the neural function is not clear in our study. Further research is warranted to address the possible explanation.

Inspired by the finding that EECP can improve ventricular-vascular coupling (Nichols et al., 2006; Beck et al., 2015), we proposed a plausible interpretation of our results, that is, an enhanced neural representation of the heart reflects the brain-heart coupling and could be modulated by EECP stimuli. Specifically, we found a significant cluster of HEP in the time window 354–389 ms after T-peak in stage EECP-1 and a significant cluster of HEP in the time window 367–387 ms after T-peak in stage EECP-2. The two significant time windows in the two consecutive EECP intervention processes mostly overlap, and this verified the robustness and reliability of EECP modulating HEP. The underlying physiological and neural mechanisms of HEP have not been completely revealed. However, baroreceptors, cardiac afferent neurons, somatosensory mapping neuro-vascular coupling and neural structures are potential pathways underlying the HEP based on current knowledge (Park and Blanke, 2019; Coll et al., 2021). We speculated that the immediate effect of EECP on HEP is not limited to affecting the heart or brain, but it is likely to change the physiological pathways and coupling strength between the brain and heart. The enhancement of HEP evoked by EECP stimuli reflects a strengthened brain-heart coupling, which increases the influence of the central autonomic network and contributes to optimal functioning. In addition, the latest studies have investigated the functional roles of HEP in pathogenesis, mental processes and prognosis, thus providing preliminary evidence for experimental and theoretical suggestions highlighting the importance of brain-heart coupling in clinical use (Yoris et al., 2017; Schulz et al., 2018; Mai et al., 2019; Schmitz et al., 2020; Tumati et al., 2021; Bogdány et al., 2022; Kumral et al., 2022; Poppa et al., 2022; Zaccaro et al., 2022). The HEP may hold promise as a biomarker in efficacy prediction for EECP therapy, however, this should be validated systematically in future studies.

There are several limitations exist within the present study. First, the recruited subjects are highly qualified physically active students with a small sample size, and this would be an obstacle to the generalization of our results. Second, the open-label study was conducted on healthy subjects, it is unknown whether EECP has a similar HEP effect in patients. Moreover, the sham method (intervention pressure 0 MPa) to serve as a placebo control in our study is imperfect. The study would have benefitted from the cuff pressure setting at a certain value (enough to feel an EECP intervention, but insufficient to alter the subject’s blood pressure.). Third, the single-channel frontal EEG cannot be used for source space and topographical similarity analyses, and it is difficult to locate the anatomical structure of the brain involved in the immediate effects of EECP on HEP. In addition, it is also very restricted to extend the effects of EECP on HEP at Fpz electrode position to the frontal lobe. Fourth, we observed the immediate effects of EECP. The sustained or long-term effects of EECP stimuli on HEP characteristics and their relation to clinical efficacy were not determined.

## 5 Conclusion

The HEP measuring neural responses to cardiac activity are sensitive to acute EECP stimuli. Our results provide preliminary evidence for the potential association between brain-heart coupling and EECP intervention. Furthermore, these findings suggest that not only hemodynamic and vascular biological effects but also the neural response to heartbeats or ANS-CNS processing might be involved in the fundamental mechanism of EECP. The brain-heart coupling quantified by HEP may serve as a feedback control parameter for the optimization of EECP devices and technology. On the premise of validation, the HEP is also likely to be used as a non-invasive and objective biomarker for the effects of EECP and the prediction of EECP treatment in cardio-cerebrovascular diseases.

## Data Availability

The raw data supporting the conclusion of this article will be made available by the authors, without undue reservation.
