# Supplementary material for: Enhanced external counterpulsation modulates the heartbeat evoked potential
Source: Front Physiol. 2023 Apr 3;14:1144073. doi: 10.3389/fphys.2023.1144073 (PMC10106756; doi:10.3389/fphys.2023.1144073)
Supplement: Supplementary file 1 [file Table1.DOCX]

Supplementary Material

**Table S1:** Two-way ANOVA results of the mean amplitude of significant heartbeat evoked potential (HEP) clusters. (effect size η_p_^2^: partial eta-squared). The intervention factor refers to active EECP or sham EECP, while the intervention phase factor refers to Baseline, EECP-1, EECP-2 and Post-EECP stages.

|  | **Intervention factor** | | | **Intervention phase factor** | | | **Intervention** $\times$**Intervention phase** | | |
| --- | --- | --- | --- | --- | --- | --- | --- | --- | --- |
| **Variable** | **F** | **P** | η_p_^2^ | **F** | **P** | η_p_^2^ | **F** | **P** | η_p_^2^ |
| **Cluster 1**  (155-169) ms | 3.545 | 0.067 | 0.085 | 3.342 | 0.046 | 0.081 | 1.476 | 0.236 | 0.037 |
| **Cluster 2**  (367-387) ms | 11.311 | **0.002** | 0.229 | 3.966 | **0.022** | 0.094 | 2.638 | 0.077 | 0.065 |
| **Cluster 3**  (354-389) ms | 11.244 | **0.002** | 0.228 | 4.114 | **0.017** | 0.098 | 3.186 | **0.043** | 0.077 |

**Table S2:** Two-way ANOVA results of the power spectral density for heart rate variability (HRV) and electroencephalography (EEG). (effect size η_p_^2^: partial eta-squared).

|  | **Intervention factor** | | | **Intervention phase factor** | | | **Intervention** $\times$**Intervention phase** | | |
| --- | --- | --- | --- | --- | --- | --- | --- | --- | --- |
| **Variable** | **F** | **P** | η_p_^2^ | **F** | **P** | η_p_^2^ | **F** | **P** | η_p_^2^ |
| **LF** | 0.011 | 0.917 | 0.000 | 3.388 | 0.074 | 0.082 | 0.188 | 0.862 | 0.005 |
| **HF** | 1.594 | 0.214 | 0.040 | 2.126 | 0.121 | 0.053 | 1.289 | 0.282 | 0.033 |
| **LF/HF** | 0.183 | 0.671 | 0.005 | 2.056 | 0.160 | 0.051 | 2.063 | 0.130 | 0.051 |
| **δ** | 0.320 | 0.575 | 0.008 | 2.272 | 0.125 | 0.056 | 2.279 | 0.124 | 0.057 |
| **θ** | 1.367 | 0.250 | 0.035 | 0.846 | 0.464 | 0.022 | 1.836 | 0.150 | 0.046 |
| **α** | 4.611 | **0.038** | 0.108 | 0.459 | 0.711 | 0.012 | 0.543 | 0.570 | 0.014 |
| **β** | 4.017 | 0.052 | 0.096 | 0.447 | 0.619 | 0.012 | 0.548 | 0.561 | 0.014 |
| **α/β** | 0.411 | 0.525 | 0.011 | 4.547 | **0.009** | 0.107 | 1.882 | 0.151 | 0.047 |

LF, low frequency (HRV frequency band 0.04-0.15 Hz); HF, high frequency (HRV frequency band 0.15-0.40 Hz); δ, Delta frequency band of EEG (0.5-4) Hz; θ, Theta frequency band of EEG (4-7) Hz; α, Alpha frequency band of EEG (8-13) Hz; β, Beta frequency band of EEG (14-30) Hz.

**Table S3:** Two-way ANOVA results of the hemodynamic parameters. (effect size η_p_^2^: partial eta-squared).

|  | **Intervention factor** | | | **Intervention phase factor** | | | **Intervention** $\times$**Intervention phase** | | |
| --- | --- | --- | --- | --- | --- | --- | --- | --- | --- |
| **Variable** | **F** | **P** | η_p_^2^ | **F** | **P** | η_p_^2^ | **F** | **P** | η_p_^2^ |
| **SBP** | 0.564 | 0.457 | 0.015 | 2.746 | 0.058 | 0.067 | 0.923 | 0.417 | 0.024 |
| **DBP** | 0.294 | 0.591 | 0.008 | 5.381 | **0.003** | 0.124 | 0.992 | 0.390 | 0.025 |
| **MAP** | 0.215 | 0.646 | 0.006 | 3.827 | **0.016** | 0.092 | 0.827 | 0.482 | 0.021 |
| **CO** | 4.168 | **0.048** | 0.099 | 2.388 | 0.083 | 0.059 | 2.805 | 0.052 | 0.069 |
| **CI** | 4.949 | **0.032** | 0.115 | 2.195 | 0.103 | 0.055 | 3.085 | **0.038** | 0.075 |
| **SVV** | 14.191 | **0.001** | 0.272 | 14.598 | **<0.000** | 0.278 | 12.726 | **<0.000** | 0.251 |
| **SVR** | 1.504 | 0.228 | 0.038 | 2.254 | 0.093 | 0.056 | 1.910 | 0.139 | 0.048 |

SBP, systolic blood pressure; DBP, diastolic blood pressure; MAP, mean arterial pressure; CO, cardiac output; CI, cardiac index; SVV, stroke volume variability; SVR, systemic vascular resistance.

**Table S4:** Correlation analysis of changes in heartbeat evoked potential (HEP) amplitude and variations of physiological parameters.

|  | **ΔHEP_1_** | | | | **ΔHEP_2_** | | | |
| --- | --- | --- | --- | --- | --- | --- | --- | --- |
|  | Active EECP | | Sham EECP | | Active EECP | | Sham EECP | |
| **Variable** | **rho** | **P_FDR_** | **rho** | **P_FDR_** | **rho** | **P_FDR_** | **rho** | **P_FDR_** |
| **Δα_1_** | 0.256 | 0.658 | 0.100 | 0.808 | 0.452 | 0.160 | -0.211 | 0.585 |
| **Δα/β_1_** | 0.434 | 0.245 | 0.206 | 0.663 | 0.118 | 0.610 | -0.023 | 0.926 |
| **ΔCO_1_** | -0.144 | 0.867 | -0.264 | 0.663 | 0.400 | 0.160 | 0.242 | 0.585 |
| **ΔCI_1_** | -0.088 | 0.867 | -0.275 | 0.663 | 0.373 | 0.160 | 0.177 | 0.585 |
| **ΔSVV_1_** | 0.039 | 0.867 | -0.060 | 0.808 | 0.318 | 0.200 | 0.189 | 0.585 |
| **Δα_2_** | 0.452 | 0.160 | -0.211 | 0.585 | 0.390 | 0.137 | -0.253 | 0.663 |
| **Δα/β_2_** | 0.118 | 0.610 | -0.023 | 0.926 | 0.155 | 0.504 | -0.047 | 0.847 |
| **ΔCO_2_** | 0.400 | 0.160 | 0.242 | 0.585 | 0.388 | 0.137 | 0.260 | 0.663 |
| **ΔCI_2_** | 0.373 | 0.160 | 0.177 | 0.585 | 0.338 | 0.168 | 0.192 | 0.663 |
| **ΔSVV_2_** | 0.318 | 0.200 | 0.189 | 0.585 | 0.417 | 0.137 | 0.154 | 0.663 |

Δvariable_1_ and Δvariable_2_ refer to the changes of variable from baseline to EECP-1 and EECP-2 stages, respectively. α, Alpha frequency band of EEG (8-13) Hz; α/β, power ratio of Alpha and Beta frequency bands; CO, cardiac output; CI, cardiac index; SVV, stroke volume variability. ΔHEP are changes in HEP amplitude of significant epochs from baseline to EECP-1 and EECP-2 stages

**Figure S1**. The original and preprocessed continuous ECG and EEG signals in sham EECP and active EECP conditions. An example of **(A)** ECG and **(C)** EEG signals of a subject from sham EECP group; **(B)** ECG and **(D)** EEG signals of a subject from active EECP group.

**Figure S2**. Raw ECG and EEG signals after 50 Hz notch filtering in EECP condition. **(A)** Raw ECG signal; **(B)** ECG signal after 50 Hz notch filtering; **(C)** Raw EEG signal; **(D)** EEG signal after 50 Hz notch filtering.

**Figure S3**. Results of discrete wavelet decomposition with Daubechies db4.


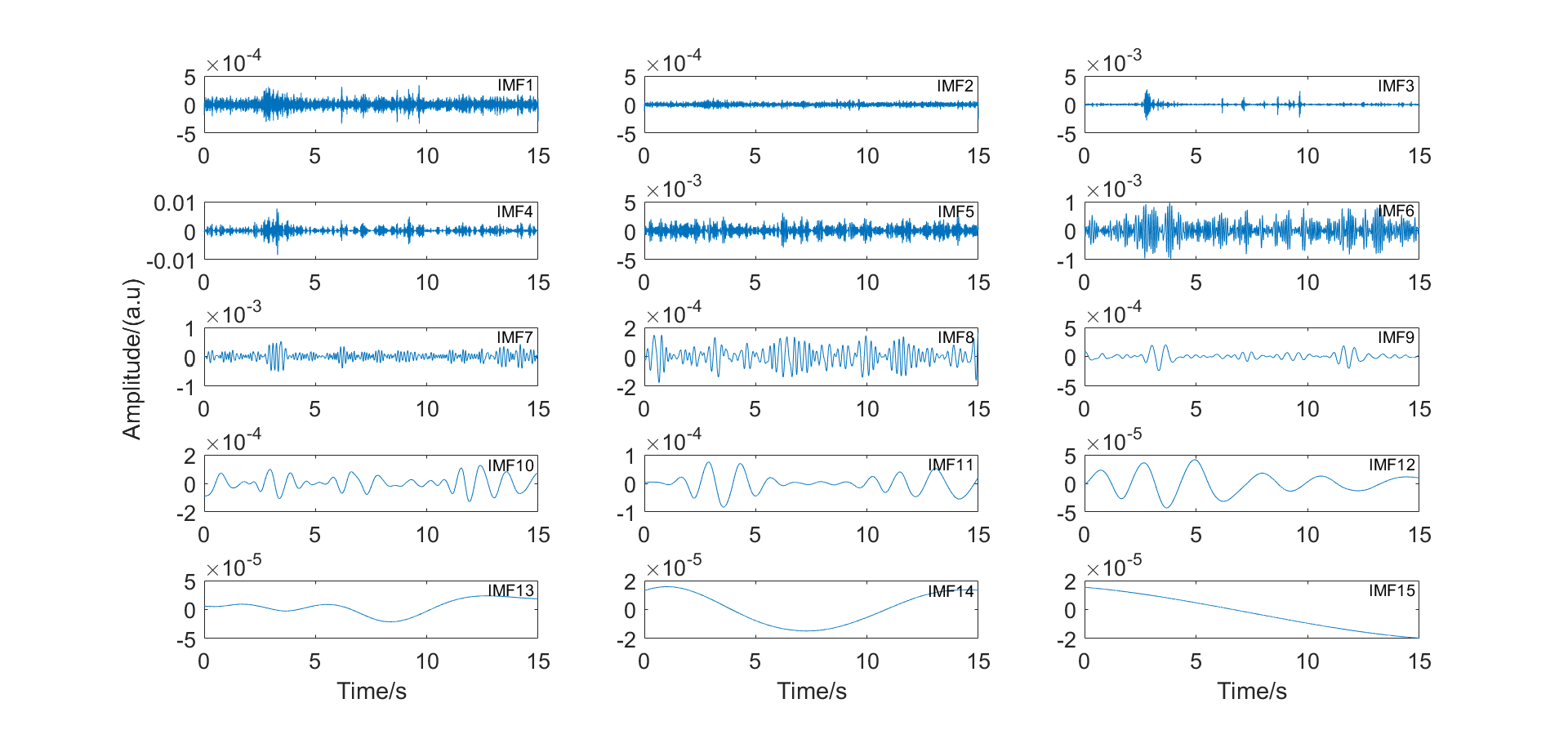


**Figure S4**. Results of complete empirical mode decomposition for adaptive noise on detail coefficient D4.


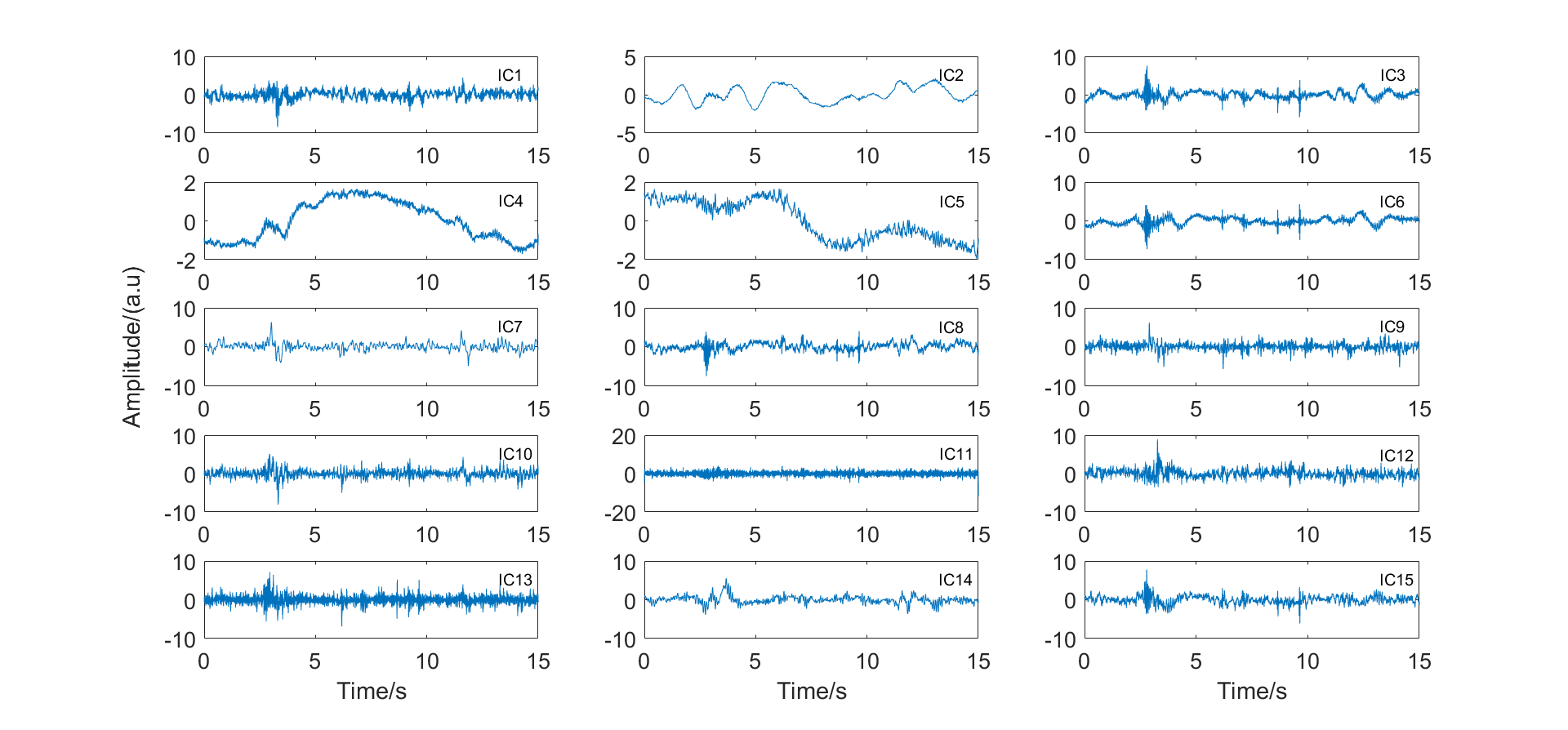


**Figure S5**. Results of ICA for IMFs of D4.

**Figure S6**. Results of signal preprocessing. **(A)** raw ECG and ECG signal preprocessed with 50 Hz notch filtering; **(B)** wavelet coefficient D4 and D4 after artifacts rejection; **(C)** raw EEG and EEG signal preprocessed by discrete wavelet transform, complete empirical mode decomposition for adaptive noise and independent component analysis.
